# Subacute High-Grade Ulnar Neuropathy Without Trauma: A Case of Cubital Tunnel Syndrome

**DOI:** 10.7759/cureus.101028

**Published:** 2026-01-07

**Authors:** Suleman Janjua, Wardha Shabbir, Andrew Lew, Nicholas Moore, Zaara Iqbal, Tariq M Awan

**Affiliations:** 1 Sports Medicine, STEM Health, Frankenmuth, USA; 2 Sports Medicine, Henry Ford Providence-Southfield Sports Medicine Fellowship, Southfield, USA; 3 Family Medicine, University of Nevada, Reno, Reno, USA; 4 Sports Medicine, Henry Ford, Southfield, USA; 5 Medical Student, University of Michigan School of Medicine, Dearborn, USA

**Keywords:** atraumatic, cubital tunnel, cubital tunnel syndrome, subacute, ulnar claw hand, ulnar nerve compression, ulnar nerve entrapment

## Abstract

Cubital tunnel syndrome (CuTS) typically presents gradually, and an acute overnight onset of severe ulnar neuropathy is rare. A 46-year-old female recreational pickleball player awoke with a right-hand claw deformity and sensory loss without preceding symptoms or trauma. Examination showed intrinsic weakness and decreased ulnar sensation; electrodiagnostic studies revealed subacute on chronic axonal and demyelinating changes at the elbow consistent with moderate CuTS. She underwent cubital tunnel release one week after onset, with rapid sensory improvement but delayed motor recovery. This case highlights an atypical acute presentation of CuTS and underscores the importance of prompt evaluation and timely intervention to mitigate persistent deficits.

## Introduction

Cubital tunnel syndrome (CuTS) is the second most common upper extremity compressive neuropathy and results from irritation or compression at any point of the ulnar nerve as it passes through from the shoulder into the hand [1,2]. Patients typically present with gradually progressive numbness and tingling of the fourth and fifth digits, often worse at night, with motor weakness and intrinsic muscle atrophy developing only in more chronic or advanced cases. CuTS is often attributed to repetitive elbow flexion, prolonged external compression usually during sleep, valgus stress from athletic activity, or prior elbow trauma [3]. Diagnosis relies on clinical examination supported by electrodiagnostic studies, and most patients initially improve with conservative management such as night splinting and activity modification [4-7]. Surgical decompression is utilized for patients with persistent symptoms or objective motor deficits [2,8].

Acute or overnight onset of severe ulnar neuropathy is uncommon and usually associated with trauma, prolonged pressure, or high-grade nerve injury. Sudden awakening with a fixed claw hand and profound motor weakness is particularly rare and not characteristic of the typical course of CuTS. We present a case of a previously asymptomatic 46-year-old recreational pickleball player who awoke with an abrupt ulnar palsy and claw hand deformity. This case illustrates an unusual, high-grade acute presentation that underscores the importance of rapid evaluation and early intervention.

## Case presentation

A 46-year-old female recreational pickleball player presented after waking up one morning with a right-hand claw deformity and decreased sensation in the ulnar nerve distribution. She had been completely asymptomatic prior to symptom onset, with no numbness, tingling, weakness, or pain, and reported no recent trauma. She had no prior history of CuTS or other neuropathies. On evaluation, she had a weak grasp and claw hand deformity with decreased sensation in the fourth and fifth digits of the right hand.

**Figure 1:**
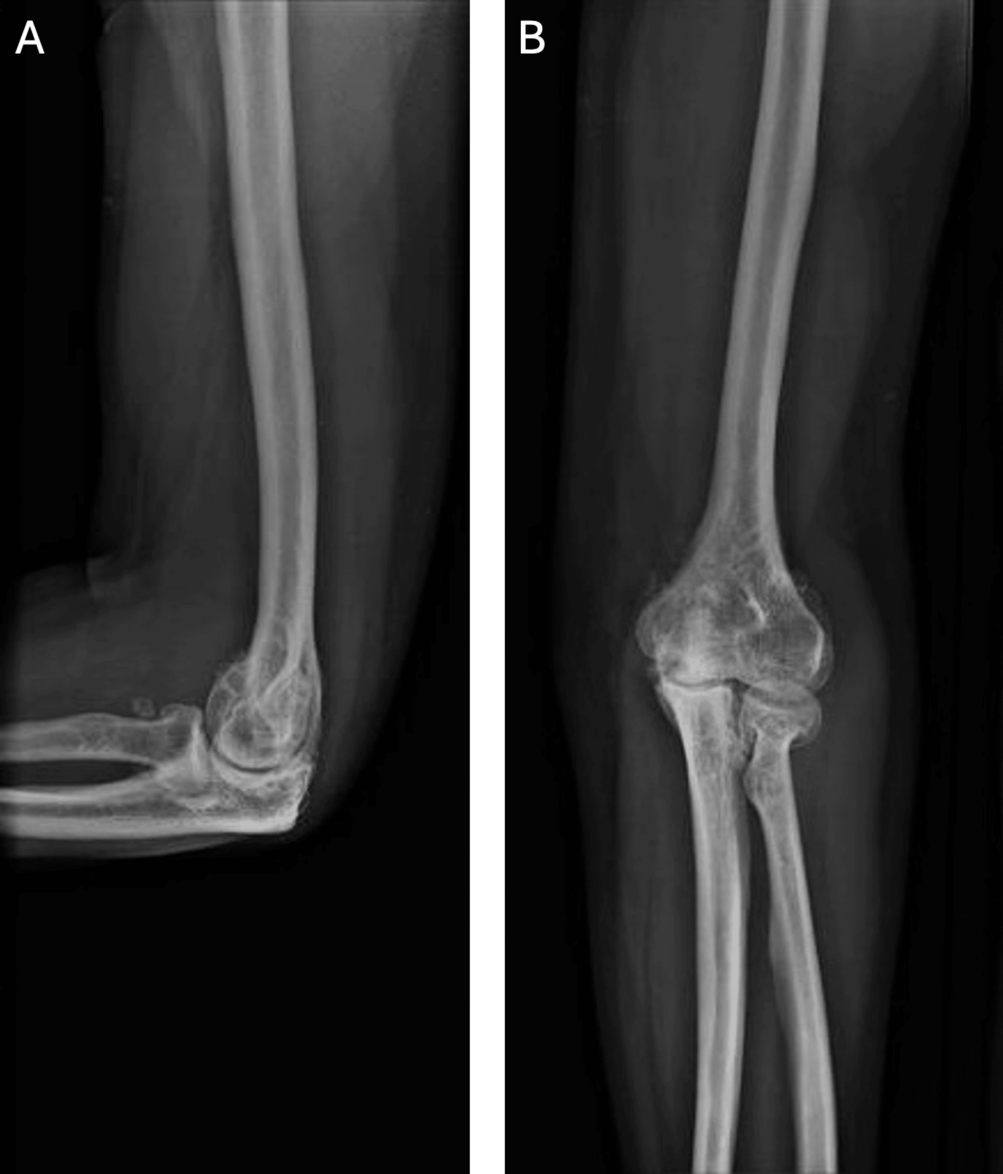
Elbow radiographs including lateral (A) and anteroposterior (B) views demonstrating mild joint space narrowing and degenerative changes without evidence of osseous deformity or acute abnormality.

On physical examination, the fourth and fifth digits of the right hand were flexed at rest at the metacarpophalangeal joint (0°) and proximal interphalangeal joint (20°). There was no erythema, swelling, warmth, or mass. Muscle strength testing revealed 5/5 extension, 4/5 flexion, 4/5 thumb adduction, and 4/5 pincer strength. Sensory examination demonstrated decreased sensation in the ulnar nerve distribution, while the median and radial nerve distributions were intact. Special tests, including Tinel’s, elbow flexion, pressure provocation, and pressure-flexion, were positive. Radiographs of the right elbow were unremarkable (Figure 1). Examination of the left hand was unremarkable.

**Figure 2:**
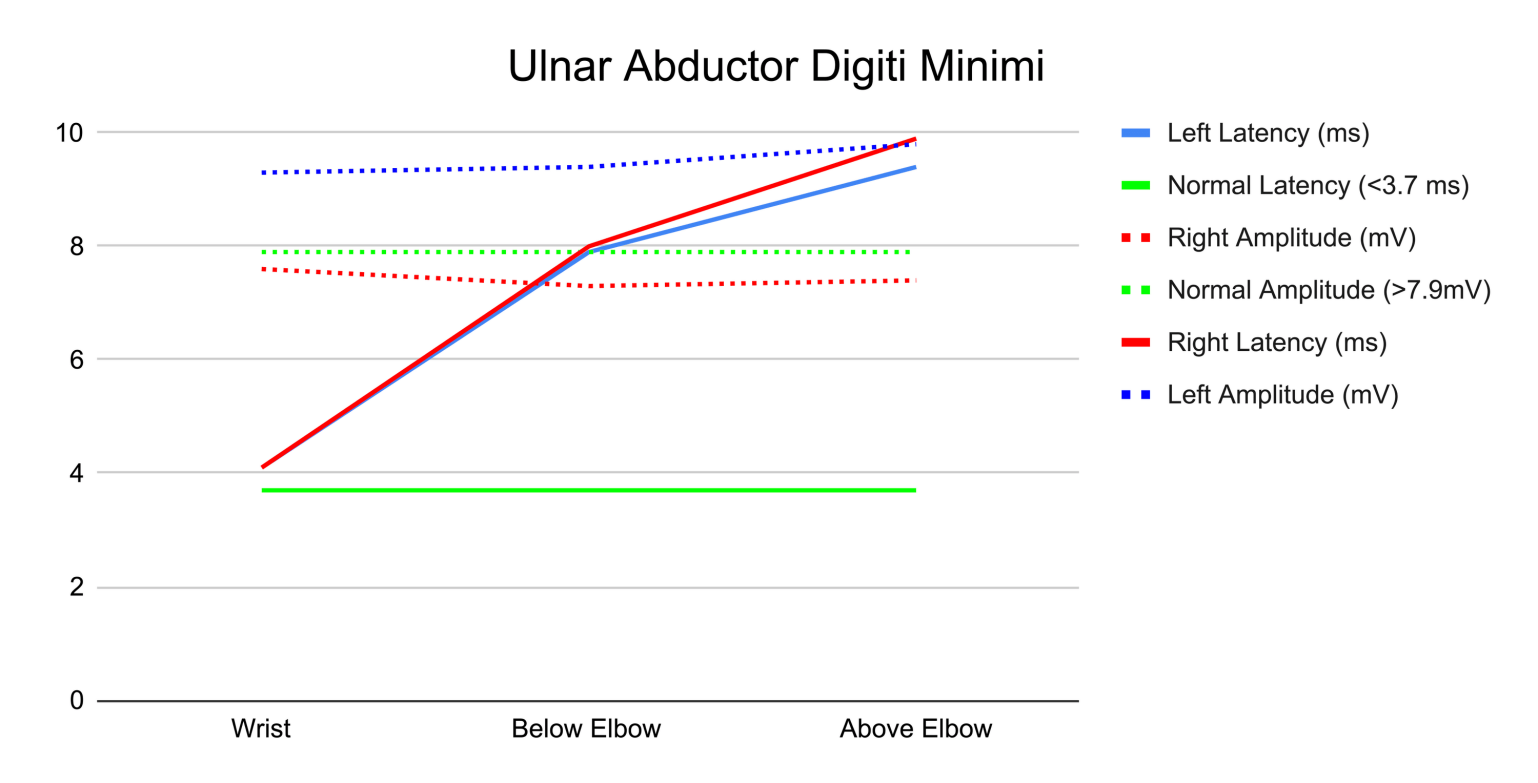
Electromyography of the bilateral ulnar nerve abductor digiti minimi. Right side in red, left side in blue, normal values in green.

**Figure 3:**
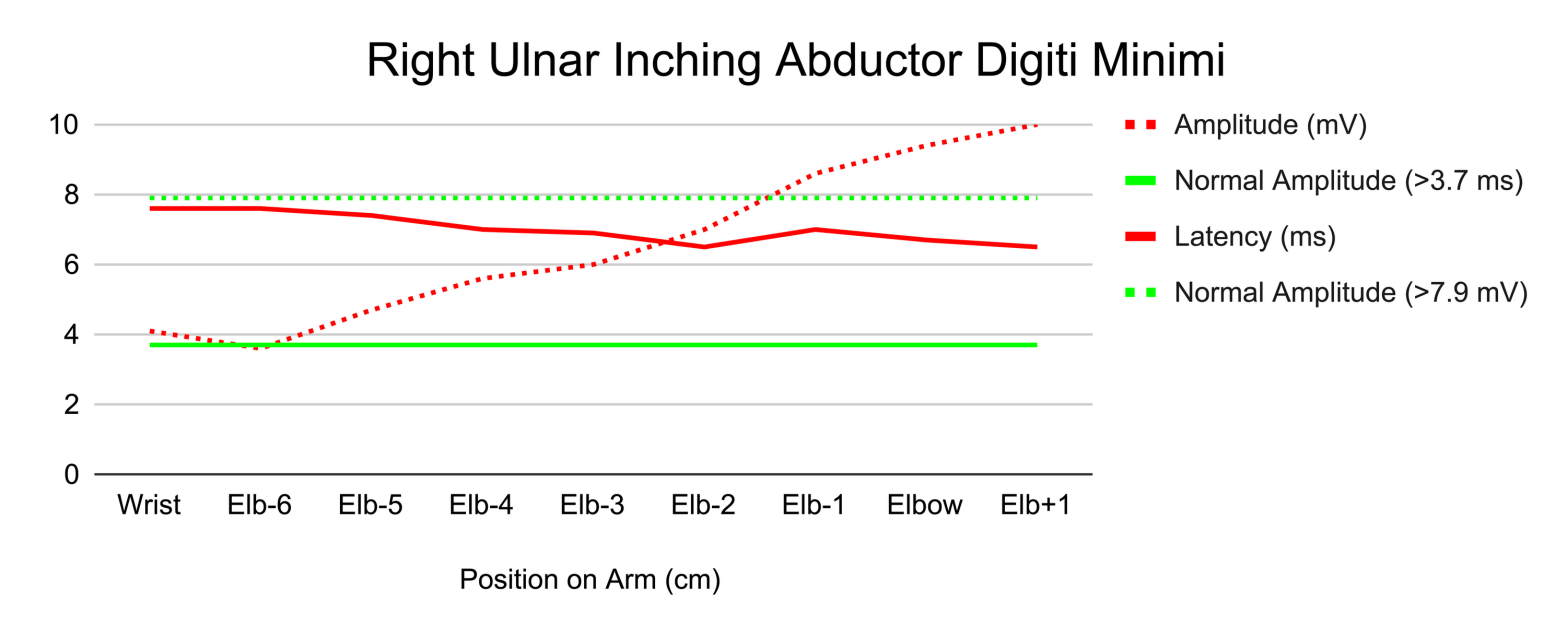
Electromyography results of the right ulnar abductor digiti minimi.

Differential diagnoses considered included Guyon’s canal syndrome, cervical radiculopathy, acute compartment syndrome, peripheral neuritis, and brachial plexus compression. Electrodiagnostic studies revealed subacute on chronic axonal and demyelinating changes of the right ulnar nerve at the elbow, consistent with moderate CuTS with active denervation and early regeneration (Figures 2-3).

The patient desired immediate relief and treatment. Given her symptoms and evidence of high-grade nerve compression, right cubital tunnel release was performed one week after symptom onset. Sensory function returned within one week postoperatively, while motor recovery remained incomplete at six months, with intrinsic hand strength not fully restored. She was subsequently cleared for a gradual return to recreational pickleball with activity modification.

## Discussion

This case illustrates an unusual presentation of CuTS, characterized by a sudden overnight onset of a fixed claw hand and severe motor deficit. Acute, high-grade ulnar nerve compression may occur due to sleep-related prolonged elbow flexion, traction injury, direct compression, anomalous anatomy, or transient ischemia.

Recreational activities, such as pickleball, may contribute to repetitive valgus stress or subtle microtrauma, predisposing the nerve to acute decompensation even in the absence of preceding symptoms [3]. In recreational athletes, such as this patient, inadequate conditioning with extended play sessions or consecutive days may aggravate overuse injuries and muscle strains, which may have led to the development of these symptoms. Using paddles that are too heavy, incorrect grip size, or employing incorrect form (i.e., relying on the wrist for backhand shots) can place excessive and improper strain on forearm muscles and elbow tendons, contributing to injury development. Additionally, though not described in this patient, inflammation from elbow tendinitis can be exacerbated by sleeping with the elbow bent for prolonged periods, leading to waking up with pain. This is a recognized trigger for acute exacerbation of existing elbow conditions, regardless of the initial cause.

The abrupt development of intrinsic muscle weakness and clawing suggests a high-grade conduction block or axonotmesis-level injury. Electrodiagnostic studies performed within days of symptom onset confirmed severe ulnar neuropathy with both motor and sensory involvement. Early surgical decompression is recommended for patients presenting with severe motor deficits or fixed deformity, as delaying intervention may limit motor recovery due to ongoing axonal degeneration [2,4,8]. In this patient, the patient sought immediate relief, and the cubital tunnel release was performed within one week of her symptom onset. The cubital tunnel release is performed by a straight or curved skin incision overlying the medial epicondyle and releasing the tensed ligaments (i.e., Osborne’s fascia), and on occasion, to transpose the nerve or resect a part of the medial epicondyle to encourage free movement and reduce symptoms.

The cubital tunnel release likely facilitated rapid sensory recovery; however, motor recovery remained incomplete at six months, consistent with the prolonged timeline of intrinsic muscle reinnervation. Conservative management of CuTS typically includes activity modification, avoidance of prolonged elbow flexion or repetitive motions, nighttime splinting, nonsteroidal anti-inflammatory medications, corticosteroid injections, and hand therapy incorporating gentle stretching and tendon gliding exercises [5]. Physical therapy modalities such as ice or heat may also be used for symptomatic relief.

This case emphasizes several clinical considerations. Acute onset of CuTS should be considered in patients who awaken with new ulnar-distribution deficits, even without trauma or prior symptoms. Early recognition and timely surgical referral are essential for optimizing outcomes. Additionally, clinicians should recognize that even recreational sports participation may increase nerve vulnerability. Injury prevention strategies emphasize a gradual increase in play intensity and frequency, utilizing proper technique and equipment, adequate rest and recovery, and proper strength and flexibility training.

Overall, this report adds to the limited literature on acute, overnight-onset ulnar nerve palsy and highlights the importance of prompt diagnosis and intervention to maximize sensory recovery, while motor recovery remains dependent on initial axonal injury severity.

## Conclusions

CuTS is common yet often overlooked until nerve dysfunction appears. Acute presentations are uncommon, typically traumatic, and rarely follow minor activity. Because compression can occur at multiple sites, accurate localization is essential. In this case, nerve conduction studies aided in identifying the suspected site of compression and guiding the decision for cubital tunnel release. Proper diagnosis and prompt intervention are crucial for recovery.
